#  Evaluation of serum HBV viral load, transaminases and histological features in chronic HBeAg-negative hepatitis B patients 

**Published:** 2017

**Authors:** Abbas Esmaeelzadeh, Hassan Saadatnia, Bahram Memar, Elham Mokhtari Amirmajdi, Azita Ganji, Ladan Goshayeshi, Zahra Meshkat, Alireza Pasdar, Hassan Vosoughinia, Mohammadreza Farzanehfar, Shahrzad Tehranian, Kamran Ghaffarzadehgan, Farnood Rajabzadeh, Mitra Ahadi

**Affiliations:** 1 *Internal Medicine Department, Faculty of Medicine, Mashhad University of Medical Sciences, Mashhad, Iran*; 2 *Department of Pathology, Faculty of Medicine, Mashhad University of Medical Sciences, Mashhad, Iran*; 3 *Department of internal medicine, Mashhad Branch, Islamic Azad University, Mashhad, Iran*; 4 *Antimicrobial Resistance Research Center, Mashhad University of Medical Sciences, Mashhad, Iran*; 5 *Department of Modern Sciences and Technologies, Faculty of Medicine, Mashhad University of Medical Sciences, Mashhad, Iran*; 6 *Clinical Research Development Center, Nuclear Medicine Research Center, Mashhad University of Medical Sciences, Mashhad, Iran*; 7 *Anatomoclinical Pathologist, Razavi hospital, Mashhad, Iran*; 8 *Department of Radiology, Faculty of Medicine, Islamic Azad University-Mashhad Branch, Mashhad, Iran*

**Keywords:** HBeAg-negative, grade, stage, viral load, AST, ALT

## Abstract

**Aim:**

To evaluate the association between biochemical, virologic and histologic features in patients with HBeAg-negative chronic hepatitis B (CHB).

**Background:**

Hepatitis-B e-antigen (HBeAg)-negative is common in Iran, is progressive with poor prognosis. Therefore, it seems necessary to perform a comprehensive evaluation of different spectrum of laboratory measurements accompanying histological findings.

**Methods:**

HBeAg- negative CHB patients referring to two university hospitals during two years were enrolled. Alcohol consumption, liver mass, fatty liver and positive results of Anti HDV, Anti HCV or Anti HIV were excluded. The relationship between viral loads, liver enzymes (old and new cutoffs) and histopathological features was analyzed using descriptive and analytic statistical methods.

**Results:**

A total of 150 HBeAg-negative CHB (males=110, mean age=38.44±11.34 years) were assessed. ALT had a significant relation with the logarithm of serum HBV-DNA (P<0.0001), grade and stage on liver biopsy (P<0.001, P=0.034, respectively). Serum viral load, AST and ALT were independent predictors of histological grade, age was the only independent predictor of the stage of liver fibrosis. There was a significant relationship between serum ALT and stage of liver fibrosis (P<0.0001) when new cutoff values for ALT were considered. We found that age had a significant relation with histological grade but it showed a reverse relation with ALT levels (P=0.009).

**Conclusion:**

In HBeAg-negative CHB, AST had a better prediction for liver necrosis and inflammation. Age could be an independent predictor for liver fibrosis. New cutoff values for ALT had superiority over conventional values to identify higher risk of liver fibrosis.

## Introduction

Hepatitis B virus (HBV) infection is one of the most serious infectious diseases in the world. Approximately one million people die annually due to chronic HBV infections (1). The prevalence of chronic hepatitis B (CHB) infection is between 2-7% in Iran, because of vertical transmission (2-4). Sixty-five percent of all CHB patients are HBeAg negative in Iran (5), with the genotype D as the most common type (4). HBeAg-negative CHB is associated with the lower HBV DNA viral load, more significant intrahepatic necroinflammatory damage, more progressive disease and higher number of cirrhosis and/or hepatocellular carcinoma compared to HBeAg-positive CHB (6-11). HBeAg-negative HBV patients are more difficult to treat than HBeAg positive due to lower sustained responses (12). Therefore, early identification of chronic hepatitis and appropriate treatment could prevent serious complications such as cirrhosis and hepatocellular carcinoma (HCC) (13).

Patients with HBeAg-negative CHB can be subject to the extensive fluctuation in ALT levels but 20-30% of these histologically documented CHB patients have normal ALT levels at the time of presentation. Consequently, patients with HBeAg-negative CHB and normal liver enzymes may be misdiagnosed as inactive chronic carriers and they mistakenly receive no appropriate treatment (10).

ALT is the most common enzyme used in the evaluation of liver disease. Recently, it has been suggested that the upper limit of normal (ULN) of ALT should be lower than 30 IU/L in men and 19 IU/L in women (14, 15). Previous studies have reported that minimal increase in serum ALT levels, even within the classic normal range, were significantly associated with an increased risk of liver-related mortality in the general population (16). 

Likewise, a recent study showed that CHB patients with normal serum ALT levels were at risk of cirrhosis and hepatocellular carcinoma (15, 16). Therefore, a more reliable cutoff value of ALT activity seems extremely important. The aim of this study was to assess the serum levels of HBV-DNA and transaminases, as well as their relation to the histological features of the liver in HBeAg-negative CHB in Iranian population. We also evaluated and compared the new cutoff values of ALT levels to the conventional cutoff values.

## Materials and Methods

HBeAg- negative CHB patients who referred to the Gastroenterology and Hepatology Clinics of two main teaching hospitals at Mashhad University of Medical Sciences, Iran, between March 2010 and March 2012 were enrolled. Diagnosis of CHB was made according to the well-established criteria (1). The exclusion criteria were considered as positive tests for HBeAg, HCV antibodies, HDV antibodies, HIV infection, consumption of alcohol, abnormalities on liver ultrasound such as masses, fatty changes and infiltrative and metabolic diseases of the liver. Serum ALT, aspartate aminotransferase (AST) and alkaline phosphatase (ALP) levels were measured using a routine automated method. In addition to the conventional cutoff value of 40 IU/L for both AST and ALT, according to the instructions by the manufacturer, new ULN cutoff values of ALT (i.e., 30 IU/L in males and 19 IU/L in females) were also applied. Liver function tests, complete blood counts, prothrombin time and bilirubin levels were assayed as well as HBsAg, HBcAb, HBeAg, HBeAb and antibodies to HCV and HDV (Roche ELISA Kit, Germany).

Liver ultrasound scan was performed on all patients in order to rule out any liver masses, especially HCC.

The identification of HBV viral load was carried out by real-time (RT)-PCR using Qiagen RT-PCR (Qiagen, USA) kit and the Taqman probe. The lower detection limit of viral load was 100 copies/mL. Cycling program is summarized in table 1. 

**Table 1:** Cycling program for RT-PCR

	**Stage**	**Time (seconds)**	**Temperature (centigrade)**
**50 cycles**	Treatment with UNG (Uracil-N-glycosylase)	15	37
	Enzyme activation	10	95
	Denaturation	10	95
	Data collection	60	60

Liver biopsy was performed in patients with elevated transaminases or HBV-DNA loads greater than 2000 units per milliliter or 10000 copies per milliliter (copies = IU x 5). Expert pathologists scored liver biopsy samples based on the histology activity index (HAI).

Statistical analysis was performed with independent t-test, Pearson`s and Spearman's rank correlation coefficient (SPSS software, version 11, Chicago, IL, USA). P-values less than 0.05 were considered statistically significant. The ethics committee of Mashhad University of Medical Sciences, approved our study.

## Results

This study included 110 men (73.3%) and 40 women (26.7%). The mean age was 38±11 years (14-79 years). Mean serum ALT, AST and ALP levels were 75.6±89 IU/L (ranging from 10 to 661 IU/L), 60.5±87 IU/L (from 6 to 819 IU/L) and 189.3±74.7 IU/L (from 45 to 470 IU/L), respectively (table-2). Our analysis showed that 89 (59.7%) out of 150 patients had ALT levels above 40 IU/L. Since about 70% of study populations were male, we adjusted the level of AST and ALT for gender. There was a significant correlation between AST, ALT levels in both crude and also in the case of adjustment for gender (R= 0.7, P<0.0001).

HBV-DNA viral load levels were evaluated in 125 patients and 97 (81%) of patients had detectable serum HBV-DNA levels (>100 IU/ml). The mean HBV-DNA level was 7.7±1.5 × 10^5 ^ IU/ml, ranging from zero to 14 107 IU/ml (table-2). Sixty (44.4%) and 42 (31.1 %) patients had HBV-DNA levels above 104 IU/ml and 105 IU/ml, respectively. 

**Table 2:** Mean age and results of liver biochemical tests

	**Mean± standard deviation**	**Range**
**Age (years)**	38±11	14-79
**ALT (IU/L)**	75.6±89	10 - 661
**AST (IU/L)**	60.5±87	6 - 819
**Alp (IU/L)**	189.3±74.7	45 - 470
**HBV DNA load (IU/ml)**	(7.7±1.5) × 10^5^	(0-14) × 10^7^

We performed a liver biopsy in 72 of 150 patients (48%). Twenty patients (27%) had stage 3 or greater and 15 (21%) had grade 10 or greater according to the HAI.

There was a reverse weak relation between age and ALT levels (P=0.049, r= - 0.168). Age also showed a direct (although not strong) correlation with the grade of inflammation (P=0.009, r = 0.306) and total score of the liver disease (P=0.003, r= 0.348).

Our findings showed that female patients experienced a significantly higher grade of liver disease (P=0.014).

**AST, ALT, and HBV DNA**

There was a relation between HBV-DNA levels and AST (P<0.0001, r=0.3) and ALT (P<0.001, r= 0.4) levels. Based on our analysis, HBV-DNA levels >10^5^ IU/ml were associated with higher ALT and AST serum levels (P<0.0001 and P<0.003, respectively).

**AST and ALT levels and grade of liver histology**

There was a direct correlation between the grade of liver disease and ALT and AST levels (P=0.049, r = 0.235 and P<0.0001, r = 0.405, respectively) although this correlation was not strong. 

An ordinal multivariate analysis of the data showed that AST, ALT and viral load were independent predictors of the histological grade (AST showed a stronger correlation, r = 0.4). The t-test analysis revealed that AST levels in patients with liver histological grades ≥10, significantly differed from those with grades <10 (P < 0.023), whereas such a difference was not observed with ALT levels (P=0.104). Thus, AST levels could be a stronger indicator for inflammation and necrosis of hepatocytes compared to ALT levels.

**AST and ALT levels and stage of liver fibrosis**

The stage of liver fibrosis was correlated with AST levels (P=0.034, r = 0.25) and age (P=0.002, r = 0.37), but no association was found between the stage and ALT levels (P=0.089).

The t-test analysis showed that serum AST levels >40 IU/L were associated with the grade of inflammation (P=0.014) and total score of fibrosis (P=0.016), but without any association with the stage of liver fibrosis. Correlation of stage of liver disease with variables of this study has been summarized in table 3.

**Table 3:** Evaluation the Spearman coefficient regression in stage of disease and other variables

	**Age**	**ALT**(IU/dl)	**Grade**	**Total score**	**Viral load(IU/ml)**	**Log of viral load**	**ALP**(IU/dl)	**AST**(IU/dl)
**Spearman coefficient regression**	0.37	0.20	0.70	0.86	-0.003	0.04	-0.18	0.25
**P-value**	0.002	0.08	0.001	0.001	0.98	0.74	0.25	0.03
**Number**	70	70	71	71	57	56	41	70

**Applying new cutoff values for AST, ALT**

Considering the new cutoff values for AST and ALT levels (i.e., 30 IU/L for males and 19 IU/L for females), ALT & AST new levels showed a direct (but not strong) correlation with number of copies of HBV DNA (p-value= 0.03, r = 0.18).

New cutoff for ALT had a direct (but not strong) correlation with the stage of disease (P= 0.003, r=0.3) and in less degree AST new cutoff levels also show a correlation with the stage of disease (p=0.04, r=0.2).

## Discussion

The most remarkable finding of the present study revealed that serum AST levels and age in HBeAg-negative CHB patients had a correlation (also not strong) with the stage of liver fibrosis. Our study showed that AST could be a better predictor of the necro-inflammatory activity of the disease compared to ALT. However, ALT levels >40 IU/L, contrary to AST, had a weak correlation with viral load. Furthermore, applying new cutoff values for ALT yielded better results including some association with viral load (P=0.03) and stage of liver fibrosis (P=0.0003), while the latter was not shown to have any correlation with ALT levels when the conventional ULN was applied. 

Although there have been many different studies on the viral load and aminotransferases (17-22) or viral load and the histology of liver disease (21, 23), up to our knowledge, a set of complete laboratory markers including viral load, AST and ALT levels, histological findings have not been studied together in a single study of HBeAg-negative CHB patients.

In this study, we used the multivariate regression analysis to assess the influence of different variables including AST, ALT, viral load and age on the histology and, especially the stage of liver fibrosis. In our study, AST had some correlation with grade and stage of liver fibrosis. Therefore, AST may be a better predictor than ALT for the inflammation and necrosis of the liver parenchyma, liver fibrosis and complications of CHB. These findings are similar to those by previous studies (17) and contrary to findings by other studies that ALT levels had a significant relation to the inflammatory grade (18, 19). However, a larger sample size could result in a more reliable picture of AST levels in clinical decision making with regard to the initiation of antiviral treatment and post-therapy follow-up.

In addition, based on our data analysis, age was an independent predictor for the stage of liver fibrosis. In a recent study, Ying, et al. showed that in CHB patients with normal AST/ALT levels, the severity of inflammation and fibrosis were positively correlated with age (20). Alam, et al. considered the age as an important factor and suggested performing liver biopsy in HBeAg-negative patients over 30 years old, regardless of serum ALT levels (21). Our study supports these findings and we believe that the age is an important factor that must be taken into account whenever performing a live biopsy is regarded for the assessment of liver fibrosis in the follow-up course of HBeAg-negative CHB patients. 

In our study, serum HBV DNA levels did not correlate with AST levels, but correlated with those of ALT. These results are similar to those by previous studies that demonstrated the significant association of ALT and HBV DNA levels (16, 21-24). Cha J. et al showed that in HBeAg-negative patients, there was no correlation between serum levels of HBV DNA and AST, while serum DNA levels were correlated with ALT (17). Serum ALT levels were also shown by Kim et al. to be correlated with HBV DNA levels in 82 patients with HBeAg-negative CHB (16). Thus, ALT levels may better reflect the reproductive status of the virus, while AST levels could be used as a better indicator of the necro-inflammatory status of the liver cells and the inflammatory grade of liver disease. Although both serum ALT and AST levels are associated with the activity of hepatitis in HBV carriers, according to some studies, AST could be a better laboratory screening test than ALT in the assessment of the severity of liver injury (17). 

It has been recently suggested that the ULN for ALT should be decreased to 30 IU/L for men and 19 IU/L for women (15, 25). A study on Chinese CHB patients revealed that patients with serum ALT levels of 0.5-1 × ULN had a significantly increased risk of complications compared to the patients with serum ALT levels < 0.5 × ULN (26). Another study indicated that HBeAg-negative patients with persistently normal ALT levels were not a homogenous group and those with high to normal ALT shared some of the characteristics that have been associated with adverse long-term outcomes (24). A recent study estimated that the best cutoff values for identifying men at risk of death from liver disease were 31 IU/L for AST and 30 IU/L for ALT and also mentioned that a slightly increased but still normal aminotransferase concentration is associated with an increased risk of death from liver disease (16). Lai et al. estimated that 37% of patients with persistently normal ALT had a significant fibrosis or inflammation on liver biopsy (27).

Thus, as mentioned above, it seems that the conventional cutoff values for distinguishing inactive carriers should be revised. Assy, et al. recently designed a prospective study that followed up patients for two years. They demonstrated that the new cutoff values for ALT (30 IU/L for males and 19 IU/L for females) together with HBV-DNA levels proposed by AASLD (American Association for the Study of Liver Diseases) and NIH (National Institute of Health) seemed efficient to differentiate inactive carriers from chronic hepatitis patients (28). In a similar study on 578 HBeAg-negative CHB patients, the revised cutoff values for ALT and a HBV-DNA load ≥100,000 copies/ml had a positive predictive value of 97% and a negative predictive value of 94% in discriminating active from inactive carriers (29).

As mentioned, in the present study the revised cutoff values for ALT (30 IU/L for men and 19 IU/L for women) had a better correlation with both the fibrosis stage and the inflammation grade of the liver and with viral load. These results are similar to findings by previous Asian studies (16, 20, 25), which may be due to the smaller muscle mass found in people from Asia. Therefore, it seems justified to decrease the AST and ALT ULN to increase significantly our ability to discriminate between inactive carriers and chronic hepatitis patients. It also helps to make a better decision whenever liver biopsies are indicated. The new cutoff values for ALT were also associated with the fibrosis stage and inflammatory grade in HBeAg-negative CHB. According to recent studies, suggested new cutoff could be used in guidelines.

The limitations of our study were the small sample size, inability to take a liver biopsy from healthy carriers, the few patients` discontent with a liver biopsy and the unavailability of genotype sequencing for the identification of HBV precore and promoter variants. We recommend a larger-scale prospective study to follow up HBeAg-negative patients with high normal ALT levels for longer periods to evaluate the revised cutoff values in this group of CHB patients more efficiently.
